# How Does the CO_2_ in Supercritical State Affect the Properties of Drug-Polymer Systems, Dissolution Performance and Characteristics of Tablets Containing Bicalutamide?

**DOI:** 10.3390/ma13122848

**Published:** 2020-06-25

**Authors:** Agata Antosik-Rogóż, Joanna Szafraniec-Szczęsny, Krzysztof Chmiel, Justyna Knapik-Kowalczuk, Mateusz Kurek, Karolina Gawlak, Vittorio P. Danesi, Marian Paluch, Renata Jachowicz

**Affiliations:** 1 Department of Pharmaceutical Technology and Biopharmaceutics, Faculty of Pharmacy, Jagiellonian University Medical College, Medyczna 9, 30-688 Krakow, Poland; joanna.szafraniec@uj.edu.pl (J.S.-S.); mateusz.kurek@uj.edu.pl (M.K.); renata.jachowicz@uj.edu.pl (R.J.); 2 Department of Physical Chemistry and Electrochemistry, Faculty of Chemistry, Jagiellonian University, Gronostajowa 2, 30-387 Krakow, Poland; gawlak@chemia.uj.edu.pl; 3 Institute of Physics, Faculty of Science and Technology, University of Silesia, SMCEBI, 75 Pułku Piechoty 1a, 41-500 Chorzow, Poland; krzysztof.chmiel@smcebi.edu.pl (K.C.); justyna.knapik-kowalczuk@smcebi.edu.pl (J.K.-K.); marian.paluch@smcebi.edu.pl (M.P.); 4 Department of Drugs Sciences, University of Pavia, viale Taramelli, 12-27100 Pavia, Italy; vittoriopaolo.danesi01@universitadipavia.it

**Keywords:** Bicalutamide, Poloxamer^®^ 407, Macrogol 6000, supercritical carbon dioxide, solid dispersions, dissolution rate, amorphization

## Abstract

The increasing demand for novel drug formulations has caused the introduction of the supercritical fluid technology, CO_2_ in particular, into pharmaceutical technology as a method enabling the reduction of particle size and the formation of inclusion complexes and solid dispersions. In this paper, we describe the application of scCO_2_ in the preparation of binary systems containing poorly soluble antiandrogenic drug bicalutamide and polymeric excipients, either Macrogol 6000 or Poloxamer^®^407. The changes in the particle size and morphology were followed using scanning electron microscopy and laser diffraction measurements. Differential scanning calorimetry was applied to assess thermal properties, while X-ray powder diffractometry was used to determine the changes in the crystal structure of the systems. The dissolution of bicalutamide was also considered. Binary solid dispersions were further compressed, and the attributes of tablets were assessed. Tablets were analyzed directly after manufacturing and storage in climate chambers. The obtained results indicate that the use of supercritical CO_2_ led to the morphological changes of particles and the improvement of drug dissolution. The flowability of blends containing processed binary systems was poor; however, they were successfully compressed into tablets exhibiting enhanced drug release.

## 1. Introduction

The growing number of active pharmaceutical ingredients (APIs) are characterized by poor water solubility. Currently, more than 70% of APIs under development are considered as poorly water soluble and almost 40% present in the pharmaceutical industry possesses aqueous solubility less than 100 µg/mL. Most of them belong to the second class of the Biopharmaceutical Classification System (BCS). These compounds are characterized by low solubility and high intestinal membrane permeability [1]. Given that the oral bioavailability of active pharmaceutical ingredients depends on their solubility and/or dissolution rate, many methods of solubility improvement have been developed [2]. They include chemical modifications, such as prodrugs [3,4] or salt formation [5], physical processes as micronization or polymorph transitions, as well as the preparation of drug-carrier systems, including solid dispersions (SD) [6,7] and complexes with cyclodextrins [8,9,10,11]. Solid dispersions are defined as drug-carrier systems in which a hydrophobic active pharmaceutical ingredient is either molecularly dispersed in a hydrophilic matrix or exists as micro-fine crystals [12]. The matrix can be made from low molecular weight compounds, or more frequently, hydrophilic polymers. They provide the dissolution rate improvement, particle size reduction, reduction of agglomeration, wettability improvement that are achieved by solid dispersion [13,14]. They possess different functional groups, molecular weight, melting temperatures (T_m_), and glass transition temperatures (T_g_). They can be crystalline (poly(vinyl alcohol)), semicrystalline (poly L-lactic acid), or amorphous (polyvinylpyrrolidone). Moreover, each polymer is characterized by different abilities to maintain molecular dispersion of active substances [15]. Drug-polymer solid dispersions are formed using evaporation methods, such as spray drying, and melting methods, including extrusion. These techniques often require high temperatures, which can influence the stability of the active substance, or high amounts of solvents, which may increase product toxicity and affect the environment [16]. These undesirable effects can be avoided by using supercritical fluid technology for solid dispersion preparation. The most commonly used gas is carbon dioxide, which is a non-toxic, non-flammable and widely available substance. In the supercritical state, i.e., above the critical temperature (31.4 °C) and pressure (74 bar), it is viscous and permeable like gaseous substances, while the density is comparable with liquids. It can act as a solvent and/or as a plasticizer for many substances. Also, it is easy to remove from the product after processing, so it is possible to obtain a solvent-free product without a dedicated solvent removal process [16,17,18,19].

Currently, tablets are the most popular dosage form. This is due to the ease of administration, neutral taste, appropriate stability and low cost of manufacturing [20,21,22,23]. Given that compression is an integral part of tableting, the effect of applied the compression force on the physical stability of the drug needs to be considered, especially in the case of the compounds undergoing mechanically induced activation [24,25]. One of such is bicalutamide, a non-steroidal antiandrogen, which belongs to class II of the Biopharmaceutics Classification System, exhibiting low aqueous solubility (below 5 µg/mL) and high lipophilicity (logP = 2.92) [26]. Th drug undergoes polymorph transition (from physically stable form I to metastable form II) or amorphization upon mechanical treatment as a consequence of a need for the relaxation of stress field applied during milling [27,28]. Drug amorphization was previously described upon milling and spray drying with polyviylpyrrolidone (PVP) [29,30]. Given that glassy bicalutamide recrystallizes easily upon grinding or scratching, the effect of increased pressure on physical stability needs to be investigated deeply. Although many papers describe the effect of temperature and humidity on the stability of amorphous drugs, the effect of pressure applied to solid dispersions during tableting was rarely described [31]. Also, only a few papers describe how the tableting and storage of solid dispersions affect the dissolution performance of bicalutamide.

The polymers chosen for this study were polyethylene glycol (Macrogol 6000, PEG6000,) and Poloxamer^®^ 407 (PLX407). These excipients are commonly used for solid dispersion preparation due to their solubilizing effects, surface adsorption, and wetting enhancement. Macrogols are widely used as carriers due to their low melting point, fast solidification, and capability of forming solid drug solutions [32,33]. However, the presence of a crystalline form may result in unstable formulations and lower dissolution rates [32]. For some drug-active substances, the improvement in solubility enhancement by Poloxamer is more efficient than by Macrogols [34]. Poloxamers, also called pluronics, are amphiphilic block copolymers consisting of hydrophilic ethylene oxide blocks (EO) attached to a central polypropylene oxide unit (PO) of hydrophobic character. They are widely used as carriers, mostly due to a low melting point. Moreover, the differences in the solubility of the constituent blocks of poloxamer macromolecule lead to thermo-responsive self-assembly in an aqueous environment, which is the additional advantage for solubility enhancement [35].

In our studies, we evaluated the use of the supercritical carbon dioxide (scCO_2_) method as a way to obtain solid dispersions with bicalutamide, which were further (after mixing with excipients) compressed into tablets. The effect of tableting and storage in both normal and accelerated conditions was evaluated together with drug dissolution performance.

## 2. Materials and Methods

### 2.1. Materials

Bicalutamide (BCL, 99.3% purity, Hangzhou Hyper Chemicals Limited, Hangzhou, China) was used as an active substance: molecular formula C_18_H_14_F_4_N_2_O_4_S, molecular weight 430.4 g/mol, solubility in water 3.7 µg/mL, logP = 2.92, pKa = 12.6, melting point 196 °C. Macrogol 6000 (PEG6000, Clariant, Muttenz, Switzerland): molecular formula H(OCH_2_CH_2_)_n_OH, average n = 100, nonionic substance, melting point 65 °C, and Poloxamer^®^ 407 (PLX407, BASF, Ludwigshafen am Rhein, Germany), molecular formula HO(C_2_H_4_O)_a_(C_3_H_6_O)_b_(C_2_H_4_O)_a_H a = 101, b = 56; average molecular weight 9840–14,600 g/mol, melting point 55 °C, freely soluble polymers were used as carriers. Carbon dioxide (CO_2_; 99.99% purity, Pszczyna, Linde Gaz, Poland) was used as a supercritical fluid. Sodium lauryl sulfate (SLS; 98.8% purity; BASF) was used to prepare dissolution medium. Distilled water was used to prepare all the solutions. Cellulose microcrystalline (JRS Pharma, Rosenberg, Germany), sodium starch glycolate (DFE Pharma, Goch, Germany) and magnesium stearate (Merck, Darmstadt, Germany) were used as excipients to formulate tablets. All excipients used to formulate tablets were of pharmaceutical grade.

### 2.2. Methods

#### 2.2.1. Solid Dispersion Preparation Using Supercritical Carbon Dioxide (scCO_2_)

Bicalutamide and carriers, i.e., Macrogol 6000 or Poloxamer^®^ 407, were mixed in a 1:1 weight ratio and loaded into a high-pressure reactor BR-300 (Berghof Products + Instruments GmbH, Eningen unter Achalm, Germany) equipped with a magnetic stirrer MR Hei-Standard (Heidolph Instruments, Schwabach, Germany), a heating mantle and a CHY 700T thermometer (CHY Firemate Co., Tainan City, Taiwan). To obtain supercritical conditions, carbon dioxide was pumped and compressed into a high-pressure reactor by a syringe pump SFT-10 (Supercritical Fluid Technologies Inc., Newark, NJ, USA). The process was conducted at 50 °C and 150 bar with Macrogol 6000 as a carrier, and at 60 °C and 160 bar with Poloxamer^®^407. In both cases, the magnetic stirrer was set at 500 rpm. The process parameters were monitored during the whole operation. The total mass of powder introduced into the high-pressure reactor was equal to 2 g. After the process, solid dispersions were pulverized through 1.8 mm sieve.

#### 2.2.2. Scanning Electron Microscopy (SEM)

The morphological characterization of the samples was conducted using a Phenom Pro desktop electron microscope (Phenom World, Thermo Fisher Scientific, Waltham, MA, USA). Powdered samples were placed on the conductive adhesive tape glued to the specimen mount and measured in a holder for non-conductive samples. The acceleration voltage was equal to 10 kV. The stream of argon was used to remove the powder loosely bounded to the tape before the measurement.

#### 2.2.3. Laser Diffraction Measurements

A Malvern Mastersizer 3000 (Malvern, UK) equipped with a HydroEV unit was used to determine particle size distribution. The samples were analyzed by the wet method using cyclohexane (reflective index, RI = 1.426) as a dispersant. Fraunhofer diffraction theory was applied to find the relationship between the particle size and the light intensity distribution pattern. The reported data represents the medians (D50 values) calculated as averages from ten series of measurements of each sample and the distribution span.

#### 2.2.4. Powder X-Ray Diffraction (PXRD)

The diffraction patterns of the samples, i.e., pure substances, solid dispersions, and tablets, were registered at ambient temperature using an X-ray Rigaku Mini Flex II diffractometer (Tokyo, Japan) with a 5°/min step. Samples were scanned from 3° to 45°. Monochromatic Cu Kα radiation (λ = 1.5418 Å) was used.

#### 2.2.5. Differential Scanning Calorimetry (DSC)

The thermal properties of the solid dispersions, raw bicalutamide, and carriers were examined using a differential scanning calorimetry (DSC) 1 STARe System (Mettler-Toledo, Greifensee, Switzerland). The measuring device was equipped with an HSS8 ceramic sensor with 120 thermocouples and a liquid nitrogen cooling station. The instrument was calibrated for temperature and enthalpy using zinc and indium standards. The glass transition temperature, crystallization, and melting were determined as the midpoint of the glass transition step, the onset of the exothermic peak, and the peak of the endothermic event, respectively. The samples were measured in an aluminum crucible (40 μL). The sample mass used for DSC experiments varied between 7 to 10 mg. All measurements were carried out with and without annealing (T = 323 K; t = 10 min). The experiments were performed from 273 to 478 K, with a heating rate equal to 10 K/min.

#### 2.2.6. Preparation of Tablet Blends

The composition of each three formulations containing raw BCL or solid dispersions are presented in Section 3.3. Bicalutamide or solid dispersions were manually mixed with excipients: cellulose microcrystalline as a filler for 3 min, sodium starch glycolate as a disintegrant for 3 min and magnesium stearate as a lubricant for 1 min, in the porcelain dish.

#### 2.2.7. Tableting Process and Tablet Characterization

The compression of tablets blends was performed using a single punch tableting machine (Korsch EK0, Berlin, Germany) equipped with the round flat punches which are 8 mm in diameter; each tablet type contained 50 mg of the active substance. Punches were equipped with tensiometers connected to a computer by Esam Traveler 1 Data Acquisition System Master Unit, type 0508-S-ESA (ESA Messtechnik GmbH, München, Germany). To obtain tablets that possess satisfactory properties, the pressure force applied on a blend containing raw bicalutamide and solid dispersions was equal to 7 kN (140 MPa) and 2 kN (40 MPa), respectively.

The mass uniformity of tablets was measured based on the method described in European Pharmacopoeia (Ph. Eur.) 9.0. The hardness, thickness and diameter of 20 randomly selected tablets were measured using hardness tester apparatus (VanKel VK200, Agilent Technologies, Santa Clara, CA, USA) and expressed as a mean value with standard deviation. The friability test was performed using a Pharma Test apparatus (Pharma Test Apparatebau AG, Hainburg, Germany). The disintegration time of the tablets was measured in distilled water at 37 °C using an Electrolab ED-2 SAPO (Electrolab, Mumbai, India) disintegration apparatus at 30 strokes/min.

#### 2.2.8. Dissolution Studies

The dissolution studies were carried out using the method recommended by the Food and Drug Administration (FDA) for BCL tablets, i.e., United States Pharmacopeia (USP) paddle apparatus (II type), and the parameters, i.e., 50 rpm, 1000 mL of 1% SLS solution, 37 ± 0.5 °C. The analysis was performed in a Vision Elite 8 apparatus (Hanson Research, Chatsworth, CA, USA) equipped with a Vision G2 AutoPlus Autosampler, and an AutoFill sample collector (Hanson Research, Chatsworth, CA, USA). Samples, i.e., raw BCL, physical mixture, and solid dispersions obtained by supercritical carbon dioxide methods, containing the equivalent of 50 mg of the active substance, as well as prepared tablets, were poured into the beakers. A volume of 5 mL of the samples was withdrawn from each dissolution vessel at predefined intervals, filtered, diluted, and assayed spectrophotometrically at λ = 272 nm (UV-1800 Shimadzu, Shimadzu Corporation, Kyoto, Japan). After the collection of each sample, the dissolution medium was replaced with the same volume of pure one. The tests were carried out in triplicate, and the results represent averages with their standard deviations.

#### 2.2.9. Contact Angle Measurements

A DSA255 drop shape analyzer (Krüss, Hamburg, Germany) was used to measure the contact angles using a sessile drop technique. A droplet of distilled water of volume equal to 2 µL was deposited on the surface of prepared tablets.

#### 2.2.10. Stability Studies

Tablets were packed into PVC/Al blisters and placed in climate chambers (Memmert GmbH + Co. KG, Schwabach, Germany) and stored in either 25 °C/60% relative humidity (long-term conditions) or 40 °C/75% relative humidity (accelerated conditions). After six-month storage, tablets were analyzed for the following parameters: mass, hardness, thickness, contact angle, and dissolution rate of bicalutamide. The solid dispersion was analyzed after its preparation. The tablets’ properties were evaluated just after the preparation and after the stability studies.

## 3. Results and Discussion

### 3.1. Solid-State Characteristics of Solid Dispersions

**Figure 1:**
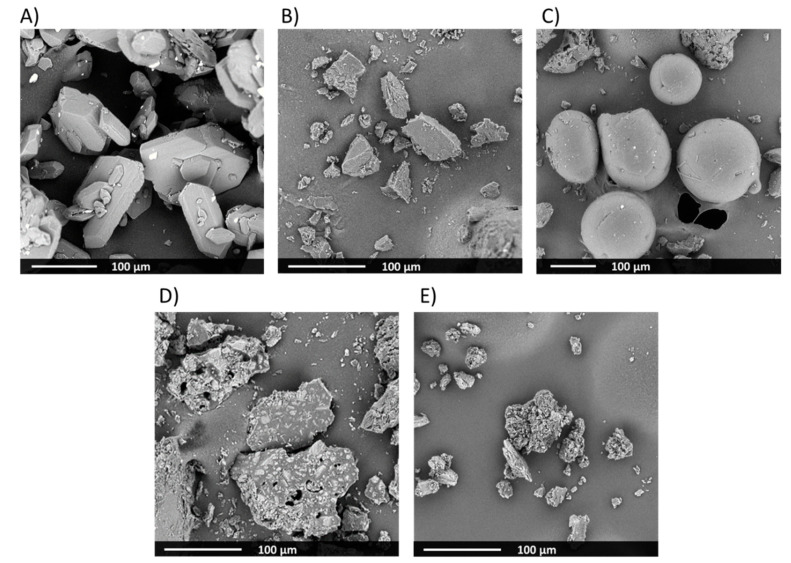
SEM image of raw bicalutamide (**A**), PEG6000 (**B**), PLX407 (**C**), BCL-PEG6000, (**D**) and BCL-PLX407 (**E**) solid dispersions.

**Figure 2:**
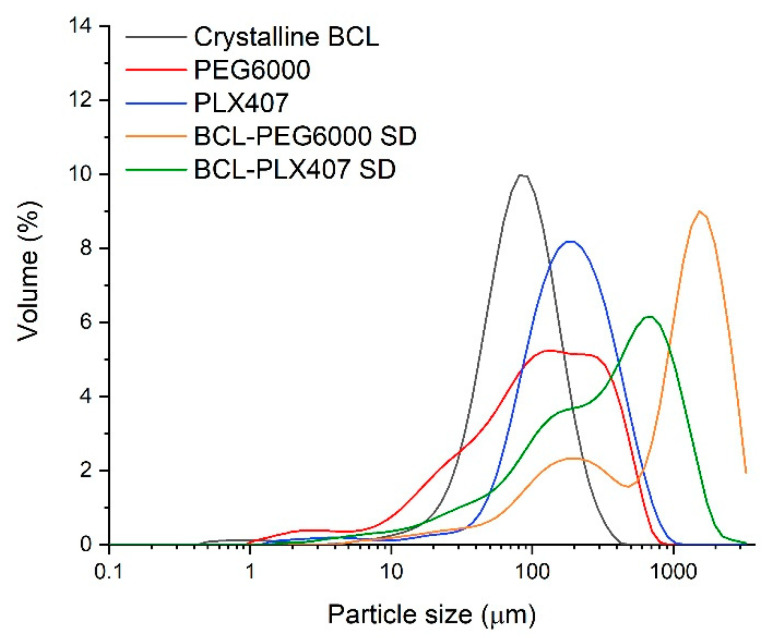
The particle size distribution of raw bicalutamide, polymers, and solid dispersions.

Based on the SEM results (Figure 1), raw bicalutamide exhibited elongated crystals of smooth surfaces. They were hexagonal and 30–160 µm long (Figure 1A). The median of the particle size distribution determined using a laser diffraction measurements laid was 81.7 µm (Table 1). The analysis of microphotographs of carriers indicated that Macrogol 6000 exhibited irregular particles of smooth surface and length not exceeding 100 µm (Figure 1B). In the case of the particles of Poloxamer^®^ 407, they were smooth and spherical, of a diameter varying between 50 µm and 250 µm (Figure 1C). The medians of the particle size were 113 µm and 180 µm for PEG6000 and PLX407, respectively (Table 1). However, the distribution span for PEG6000 was much higher than for the other compounds, which indicated a wide distribution. The analysis of the particle size distribution revealed the presence of dust particles in each sample visible as a distribution tail within the region below 10 µm (Figure 2). In the case of bicalutamide and PLX407 the distributions were narrow and symmetrical (despite said tails), while in the case of Macrogol, the particle size was distributed uneven, with a maximum shift towards big particles. The distribution curve is skewed to the left, which can explain the differences between the size calculated from the SEM picture and laser diffraction measurements. The particles visualized using an electron microscope represent the fraction described by the extended distribution shoulder visible on the left side of the plot (pictures showing greater particles at the same magnification were not representative enough and are not presented here), while the laser diffraction assessed all the particles present in the sample.

After the treatment of BCL-PEG6000 binary mixtures by a supercritical carbon dioxide at 130 bar and 50 °C in the high-pressure reactor, solid dispersions were pulverized and white, free-flowing powders were obtained. In the case of solid dispersion with Poloxamer^®^ 407, prepared at higher process parameters, i.e., pressure 140 bar, temperature 60 °C. The obtained sample also was white; however, its structure was foamy. After pulverized, white, free flowing powder was obtained.

Solid dispersions morphology was analyzed using SEM. The obtained results showed that the sample containing Macrogol 6000 exhibited irregular particles of a smooth surface with well-visible incorporated particles of different contrast. They were identified as the crystals of the active substance (Figure 1D). The particles of the solid dispersion with Poloxamer^®^ 407 were rough, with visible inclusions of BCL crystals (Figure 1E).

The laser diffraction measurements revealed that solid dispersions exhibited a wide particle size distribution, in both cases skewed to the left, with two visible modes (Figure 2). The median of BCL-PEG6000 solid dispersion particles lay above 1 mm, which could be a result of the intense laser light diffraction on big particles and the presence of a well-resolved mode of the distribution curve. In the case of solid dispersion with Poloxamer^®^ 407, the median of the particle size distribution was equal to 356 µm (Figure 2).

**Figure 3:**
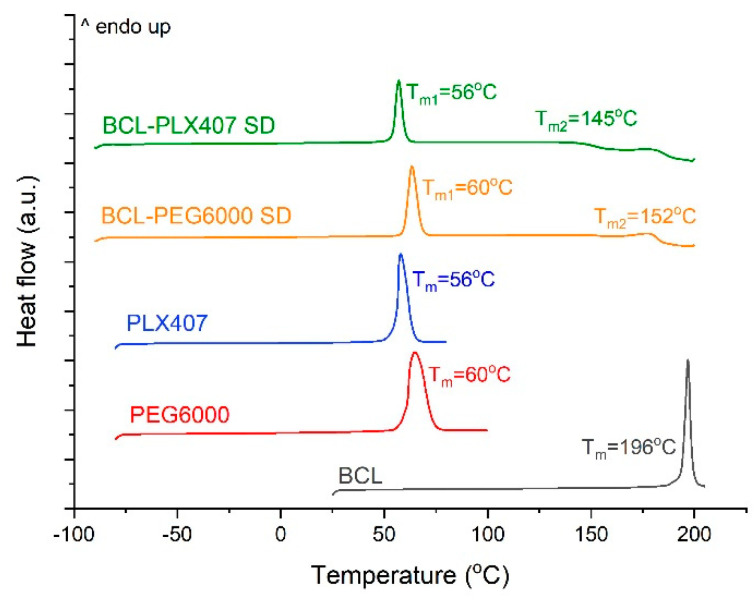
Scanning differential calorimetry of raw bicalutamide, polymers, and solid dispersions.

The thermal properties were obtained using differential scanning calorimetry (DSC). The presence of an endothermic peak with an onset at 196 °C corresponds to the melting temperature of form I polymorph of bicalutamide (Figure 3). After the treatment with supercritical CO_2_, decreases in bicalutamide crystallinity in both obtained solid dispersions were noticed. This was confirmed by a decrease in the melting temperature of the active substance. For the solid dispersion prepared with Macrogol 6000, the melting of bicalutamide was recorded as an onset at 152.6 °C. The presence of the second endothermic peak with the onset at 60 °C corresponds to the melting point of the carrier. In the case of solid dispersions with Poloxamer^®^ 407, the glass transition temperature was recorded at −6.2 °C. Bicalutamide crystallization occurred at 145.4 °C, while it melted at 167.4 °C. This indicated that the treatment with supercritical CO_2_ led to drug amorphization. However, the system was characterized by very low physical stability in ambient conditions, expressed as a negative value of T_g_. It resulted from low values of PLX407 T_g_, −65 °C, combined with T_g_ of amorphous form of bicalutamide equal to 55 °C [36,37].

**Figure 4:**
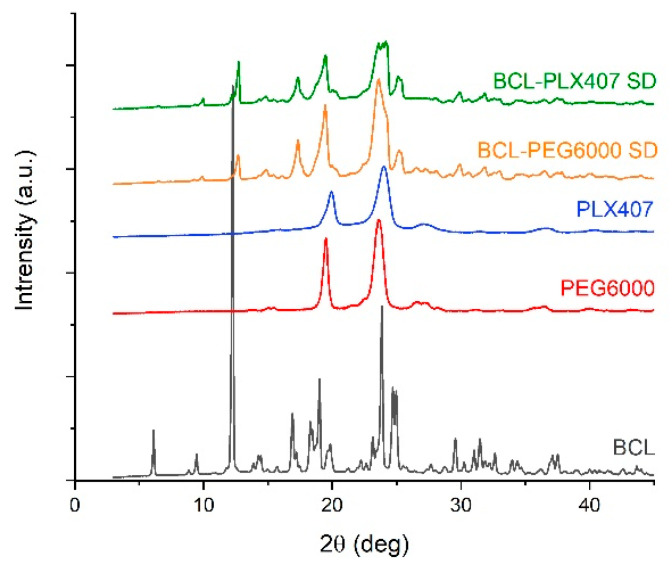
X-ray diffraction of raw bicalutamide and carriers, i.e., Macrogol 6000 and Poloxamer^®^ 407, and solid dispersions.

The molecular structure of each compound and solid dispersions was assessed using X-ray diffractometry. The diffraction pattern of raw bicalutamide is characteristic for crystalline form I polymorph, rich in distinctive Braggs peaks at 2θ equal to 6.2°, 9.5°, 12.3°, 14.3°–14.6°, 17.0°, 19.0°, 20.0°, and 24.0° (Figure 4). In the case of the used carriers, the diffraction patterns showed only two signals for each component, resulting from the semi-crystalline nature of the polymers. A decrease in the drug crystallinity was noticed in both types of solid dispersions. It was demonstrated by the decrease in the intensity and broadening of the peaks (Figure 4). Interestingly, the positions of the peaks shifted in comparison with the initial form of the drug. This indicated the recrystallization into the second polymorph of BCL, with the Braggs peaks positioned at 12.8°, 17.4°, 19.5°, 23.7–24.3°, and 25.4° [27].

### 3.2. Bicalutamide Dissolution

**Figure 5:**
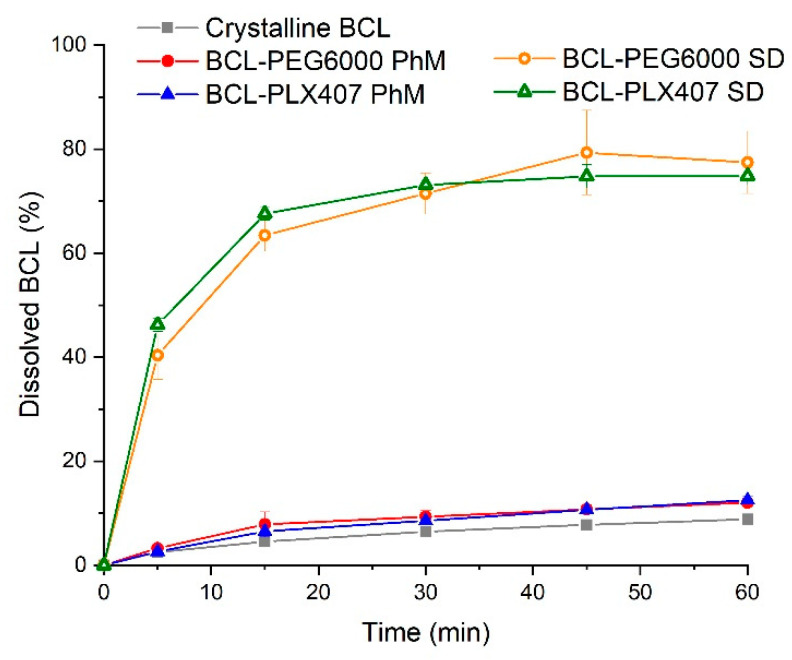
Dissolution of bicalutamide from solid dispersions with Macrogol 6000 and Poloxamer^®^407 in comparison with physical mixtures and crystalline bicalutamide (form I polymorph).

The preparation of solid dispersions using the supercritical carbon dioxide method with Macrogol 6000 and Poloxamer^®^407 led to a decrease in bicalutamide crystallinity, which resulted in dissolution improvement. The dissolution profiles of bicalutamide were very similar in both types of solid dispersions (Figure 5). After 1 h, 74.80 ± 1.66% of the drug dissolved from BCL-PEG6000 solid dispersion, and 77.43 ± 6.01% from the BCL-PLX407 one. The amount of bicalutamide dissolved from solid dispersions was approximately nine times greater than that determined for crystalline bicalutamide (form I polymorph), as 8.85 ± 1.02% of pure drug dissolved. No significant differences in the drug dissolution characteristics were noticed after the physical mixing of the active substance with any of the two polymers (approximately 12% of drug dissolved from each mixture). This confirms that the hydrophilic character of the carriers was not a crucial factor driving drug dissolution, and the dissolution improvement was related to the changes that occurred after the treatment with supercritical CO_2_. During the process, the drug substance was dissolved in the polymer in supercritical CO_2_, and the magnetic stirrer ensured uniform mixing of the ingredients. In the process of carbon dioxide removal, the sample solidified as a result of the rapid decrease in pressure. It provided homogenous distribution of the drug substance in the carrier.

### 3.3. Characteristics of Tablets Containing BCL-PEG6000, BCL-PLX407 Solid Dispersions and Raw BCL

The three types of tablets were prepared using the direct compression method. The BCL-PEG6000 and BCL-PLX407 solid dispersions and raw bicalutamide were mixed with cellulose microcrystalline, sodium starch glycolate, and magnesium stearate to formulate tablets (Table 2). The obtained results revealed that hardness, thickness and friability were satisfactory.

The analysis of hardness showed that the tablets containing solid dispersions were characterized by higher hardness than those with raw bicalutamide (Table 3). This resulted from the binding properties of the used carriers, which also affected the disintegration time. Tablets containing solid dispersions disintegrated for longer than tablets with raw bicalutamide. Moreover, the tablets with BCL-PLX407 solid dispersion exhibited a six-fold longer disintegration time than those with BCL-PEG6000 solid dispersion, 24 min 22 s and 4 min 23 s, respectively. The difference was caused by the properties of poloxamer, which behaves like a binding substance [38]. During the compression process, particles tend to aggregate and increase the number of direct contact points between them, which extends tablet disintegration. Despite the surface activity and solubilizing activity of the polymer, a great content of poloxamer in the sample can retard tablet disintegration due to the gelling properties of the polymer [38,39,40].

The use of hydrophilic polymers in solid dispersion resulted in an increase in the wettability of the surface in comparison with the tablets containing raw bicalutamide. The tablets containing solid dispersions were more hydrophilic. They exhibited lower values of contact angles than the tablets with crystalline BCL:50.8° and 55.8° for the tablets containing PEG6000 and PLX407, respectively.

**Figure 6:**
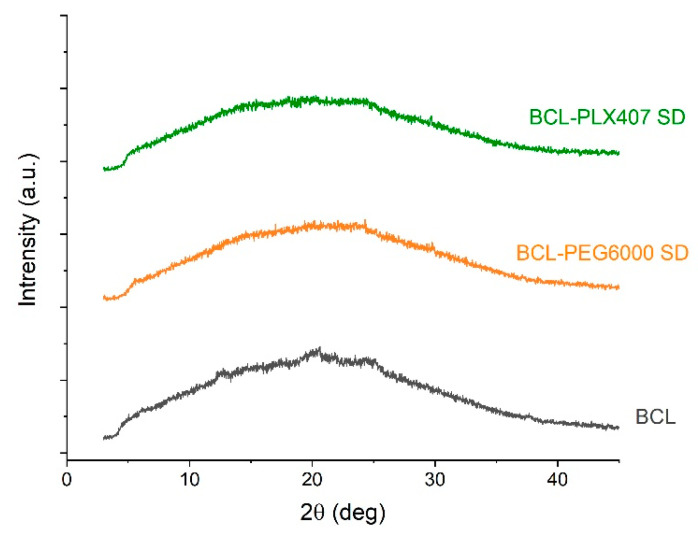
Diffraction patterns of tablets containing raw bicalutamide and solid dispersions.

The X-ray diffraction analysis indicated that the active substance was amorphous. The presence of a broad halo instead of the Braggs peaks characteristic for crystalline drug substances, visible in Figure 4, indicated that bicalutamide was amorphous in all tested tablets (Figure 6). We assumed that this resulted from the propensity of bicalutamide to undergo activation upon mechanical treatment [10,12,27]. This means that the material gains some amount of energy which needs to be released. The relaxation of the stress field, applied during compression, occurs in several ways, including amorphization, as in the case of investigated tablets.

**Figure 7:**
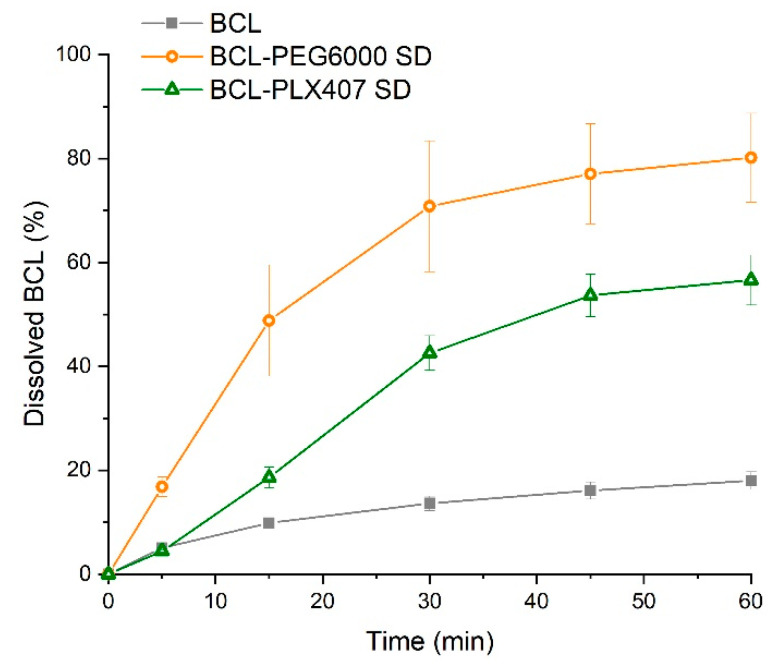
Dissolution profile of tablets containing solid dispersions and raw bicalutamide.

As shown in Figure 7, bicalutamide release from the tablets indicated that the use of solid dispersions in tablets significantly enhanced drug release. This resulted from the improved wettability and amorphization of bicalutamide upon compression. After 1 h, 80.16 ± 8.56% and 56.61 ± 4.84% of the drug released from the tablets with PEG6000 and PLX407 solid dispersions, respectively. From tablets containing raw bicalutamide, 17.99 ± 1.70% dissolved, which was two times greater in comparison with the drug alone. Although the dissolution of bicalutamide from both solid dispersions was comparable at each time point, the differences in profiles were noticeable between solid dispersions (Figure 5) and corresponding tablets. After 30 min, 28% more bicalutamide was dissolved from tablets containing BCL-PEG6000 solid dispersion in comparison with the BCL-PLX407 tablets.

The prepared tablets were evaluated for stability upon storage. The results of the studies after six months revealed that the storage in both the long-term and accelerated conditions affected the properties of the tablets (Table 4) in comparison with tablets analyzed immediately after preparation (please refer to Table 3).

**Figure 8:**
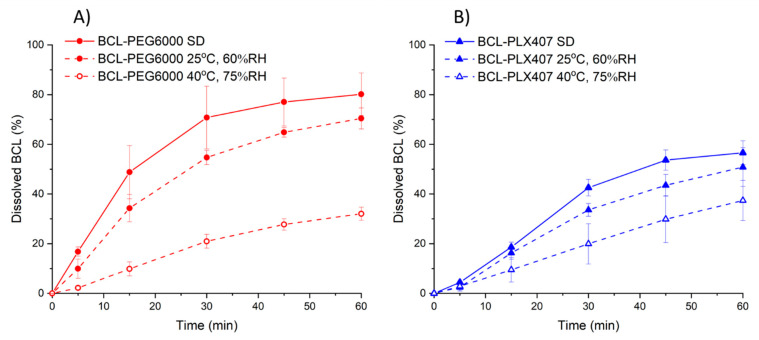
Release profiles of bicalutamide from tablets containing solid dispersion with either PEG6000 (**A**) or PLX4087 (**B**) after stability studies, in comparison with tablets analyzed after preparation.

In both types of tablets, drug release decreased after storage, regardless of the storage conditions (Figure 8A,B). However, less noticeable changes occurred for tablets stored at 25 °C and 60% RH. The amount of dissolved bicalutamide was 70.42% and 50.82% in the case of tablets containing solid dispersion with PEG6000 and PLX407, respectively. On the contrary, 32.01% of bicalutamide was released from BCL-PEG6000 tablets stored in accelerated conditions, which was a 2.5-fold decrease, in comparison with the freshly prepared tablets.

**Figure 9:**
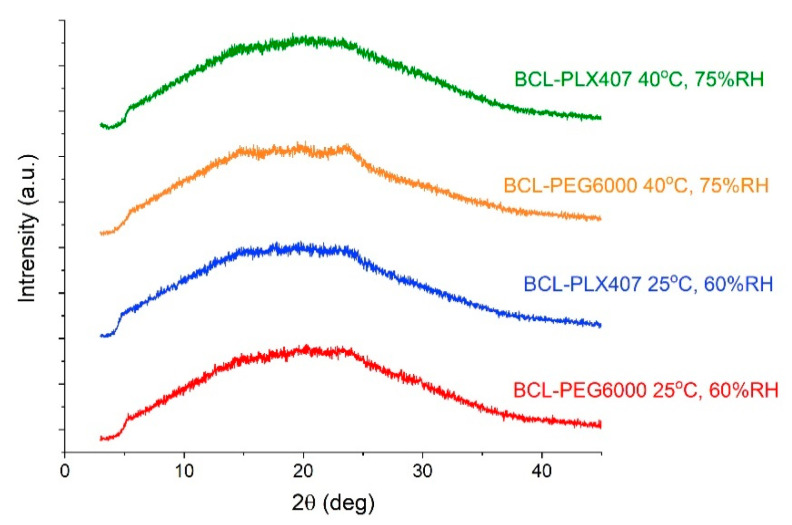
Diffraction patterns of bicalutamide in tablets with solid dispersions after stability studies.

Moreover, after the six-month stability studies, no phase transition of the drug substance occurred (Figure 9). The diffraction patterns did not change in comparison with those measured for tablets directly after compression (Figure 6). The bicalutamide remained amorphous, as confirmed by the halo visible in the diffractograms (Figure 9). This indicated that water absorbed from the air in the climate chambers did not cause drug recrystallization.

## 4. Conclusions

This study has examined the effect of supercritical carbon dioxide treatment on the properties of binary systems containing bicalutamide and Macrogol 6000 or Poloxamer^®^ 407. The dissolution and attributes of tablets were evaluated. The scCO_2_ influenced not only the size and shape of the particles of the processed substances, but also the dissolution characteristics of bicalutamide from solid dispersions. Supercritical conditions induced a decrease in the crystallinity of the active substance. The obtained solid dispersions were successfully formulated in tablets. The tableting process induced the amorphization of the drug substance. This phenomenon confirmed the sensitivity of bicalutamide on mechanical activation. After 1h, the amount of bicalutamide dissolved from tablets with PEG6000 remained unchanged. In the case of tablets containing Poloxamer^®^407, the amount of released substance decreased by 20%. However, the compression affected the dissolution profiles of the drug substance in comparison with solid dispersion. The stability studies indicated that storage conditions affected the tablets’ properties. The amount of the released drug decreased regardless of the storage conditions. However, in the stability studies after six months, the diffraction patterns did not change, which indicated that the physical state of the active substance was unchanged.

## Figures and Tables

**Table 1:** Particles size distribution of raw bicalutamide and carriers, results obtained by the laser diffraction method.

Substance	D_50_ (µm)	Span
Bicalutamide	81.7	1.70
Macrogol 6000	113.0	3.18
Poloxamer^®^ 407	180.0	2.02
BCL-PEG6000	1120	2.01
BCL-PLX407	356	2.90

**Table 2:** The composition of tablets containing BCL-PEG6000 and BCL-PLX407 solid dispersions and raw bicalutamide.

Substance	Tablets					
	Raw BCL		Solid Dispersion			
			BCL-PEG6000		BCL-PLX407	
	Content of Compounds in One Tablet					
	mg	%	mg	%	mg	%
BCL	50.00	41.67	-	-	-	-
BCL-Polymer (SD)	-	-	100.00	58.82	100.00	58.82
Cellulose	-	-	-	37.65	64.00	37.65
Microcrystalline	64.00	53.33	64.00			
Sodium starch glycolate	4.80	4.00	4.80	2.82	4.80	2.82
Magnesium stearate	1.20	1.00	1.20	0.71	1.20	0.71

**Table 3:** The properties of tablets containing solid dispersions (SD) and crystalline bicalutamide.

	Parameter	Mass (mg)	Thickness (mm)	Hardness (kp)	Friability (%)	Disintegration Time (min:s)	Contact Angle (°)
System							
SD BCL-PEG6000		169.3 ± 7.0	3.60 ± 0.04	2.87 ± 0.63	0.0	4:23	50.8
SD BCL-PLX407		168.1 ± 5.8	3.43 ± 0.04	2.28 ± 0.64	0.4	24:22	55.8
Raw BCL		121.5 ± 3.6	2.08 ± 0.04	1.97 ± 0.25	0.6	00:31	85.4

**Table 4:** Properties of tablets after six months of storage.

	Parameter	Mass (mg)	Hardness (kp)	Thickness (mm)	Disintegration Time (min:s)	Contact Angle (°)
System						
25 °C, 60% RH						
SD BCL-PEG		168.3 ± 3.5	2.47 ± 0.15	3.66 ± 0.01	04:57	48.9
SD BCL-PLX		174.5 ± 5.1	2.63 ± 0.51	3.53 ± 0.05	27:10	58.9
40 °C, 75% RH						
SD BCL-PEG		177.0 ± 4.4	1.80 ± 0.36	3.73 ± 0.08	04:59	59.8
SD BCL-PLX		175.9 ± 5.5	1.25 ± 0.07	3.76 ± 0.06	14:39	53.8
